# ‘Traumatic to Say the Least; It Still Affects Me’: Consumer Perspectives on Community Treatment Orders in Australia

**DOI:** 10.1111/hex.70770

**Published:** 2026-07-17

**Authors:** Sharon Lawn, Tessa‐May Zirnsak, T. J. Spencer, Elaine Waddell, Jane Fischer, Tarmia Klass, Puneet Sansanwal, Edwina T. Light, Vrinda Edan, Chris Maylea, Penelope Weller, Morgan Gould, Christopher J. Ryan, Giles Newton‐Howes, Steve Kisely, Lisa Brophy

**Affiliations:** ^1^ College of Medicine and Public Health Flinders University Adelaide South Australia Australia; ^2^ Lived Experience Australia Ltd Adelaide South Australia Australia; ^3^ Department of Community and Clinical Health La Trobe University Melbourne Victoria Australia; ^4^ University of Sydney Sydney New South Wales Australia; ^5^ Medicine, Dentistry and Health Sciences University of Melbourne Melbourne Victoria Australia; ^6^ Victorian Mental Illness Awareness Council (VMIAC) Melbourne Victoria Australia; ^7^ Law School, La Trobe University Melbourne Victoria Australia; ^8^ School of Law, RMIT University Melbourne Victoria Australia; ^9^ Discipline of Psychiatry and Mental Health University of New South Wales Sydney New South Wales Australia; ^10^ Department of Psychiatry St Vincent's Hospital Darlinghurst New South Wales Australia; ^11^ Department of Psychological Medicine University of Otago Wellington New Zealand; ^12^ School of Medicine University of Queensland Brisbane Queensland Australia; ^13^ Metro South Health Service Woolloongabba Queensland Australia

**Keywords:** coercion, community treatment orders, mental health, mental health services, organisational culture, severe mental illness

## Abstract

**Background:**

Community Treatment Orders (CTOs) are contested practices in mental healthcare due to unresolved evidence of effectiveness and persistent human rights concerns. Despite this, Australia has high rates of CTO use internationally. Research from a lived and living experience (LLE) perspective remains limited.

**Methods:**

This mixed methods research explored experiences of people with LLE of mental ill‐health and being on a CTO (‘consumers’). An online survey including Likert‐rated, categorical and open‐ended questions was disseminated across Australia in 2024 to consumers aged 18 years or over. Quantitative data were reported descriptively, and qualitative data were analysed using Latent Content Analysis.

**Results:**

Forty‐three people completed the survey; most identified as female (86%); 51.2% were aged 30‐49 years; 7.2% were currently on a CTO; 40.5% had been on a CTO in the past 1‐5 years; and 45.2% had been on a CTO more than once. Automated data contamination (‘bot’) infiltration hampered inclusion of further data. Participant responses included: (1) experiences prior to being on a CTO; (2) experiences whilst on a CTO; (3) attitudes towards CTOs; (4) family involvement; and (5) views on mental health services. While some consumers reported experiencing positive care, many rejected use of CTOs, reporting coercion, exclusion from decision‐making and information, lack of alternative support options, and lack of compassion.

**Conclusions:**

Policy and practice reforms, including focus on recovery and human rights, appear to have failed to improve experiences of CTOs. Further work to close the policy‐practice gap and translate policy into more inclusive, human rights‐based care is indicated.

**Lived Experience or Public Contributions:**

This project has included considerable involvement and community engagement with people with lived and living experience (‘LLE’) of CTOs and/or mental health service use in all phases of the project. The project includes two Chief Investigators who identify as LLE leaders and researchers (VE and SL), and who have direct lived experience of CTOs, or as a family/caregiver of individuals who have direct lived experience of CTOs. The project manager (TZ) and some project staff (TS, TK, and PS) are LLE researchers, and the project is guided by a Lived Experience Advisory Panel (LEAP) with LLE experience of CTOs, including both service users and family/caregivers. We also have LLE experts on our project's international advisory panel. Together, their contributions have been vital in the project team's open and transparent reflections on their diverse perspectives and positioning which have grounded the project's focus on LLE perspectives.

## 1 Background

Deinstitutionalisation has been a common feature of societal shifts in attitudes, policy reform, and mental healthcare practice in many comparable high‐income countries. It occurred as a consequence of shifting knowledge, attitudes and awareness of the needs and rights of people with mental health conditions, including the hope that shifting from incarceration in large‐scale institutions to psychiatric treatment in ‘the community’ would provide more humane recovery‐oriented shifts in treatment, care and support. Institutions were also increasingly expensive to run, and by the 1990s, when psychotropic medications also became more widely available, deinstitutionalisation was common across high‐income countries. However, with health systems, services, and communities ill‐prepared and resourced, new forms of coercive care in the community emerged in the form of Community Treatment Orders (CTOs), in response to these gaps and as an alternative to hospitalisation and other institutional treatment options [1]. These orders are implemented under statutory powers conferred by mental health legislation, which provides the legal framework for mental health treatment in the community and defines the rights of individuals and responsibilities and powers of clinicians and other authorities (see examples) [2, 3, 4, 5].

The use of CTOs, however, remains contested in mental health service delivery internationally. This arises from unresolved questions about their effectiveness [6], wide variation in their use [7, 8, 9], and ethical and human‐rights concerns [8, 9, 10, 11]. Concern about the indiscriminate use of involuntary treatment in the community is also amplified by the adoption of the United Nation Convention on the Rights of Persons with Disabilities (CRPD) [12, 13] which requires respect for equality, recognition of the capabilities of people with disabilities, and the provision of supports to enable such capabilities to be exercised. According to the World Health Organization (WHO) the CRPD calls for a scaling up community‐based services that promote more holistic person‐centred, rights‐based and recovery‐oriented services, with specific focus on respect for legal capacity, non‐coercive practices, participation, community inclusion, and a recovery‐oriented approach [14]. Within the context of the current paper, we define ‘recovery‐oriented’ as an approach that supports the person in ‘regaining control of their identity and life, having hope for their life, and living a life that has meaning for them whether through work, relationships, spirituality, community engagement or some or all of these.’ [14]^(p.5)^ We define ‘coercive care’ as any measure applied against the person's will or consent, and can include formal coercion or restrictive practices (actions limiting freedom of movement (e.g. restraint, seclusion, involuntary hospitalisation) and forced treatment (e.g., CTOs), and informal coercion (any form of influence, pressure, or manipulation of the person's decisions) [14, 15].

Within this context, Thornicroft and Henderson state, ‘the CRPD makes clear that direct decision making by patients and forms of supported decision making are permissible under the convention, but that substitute decision making (which is the essence of compulsory treatment decisions by psychiatrists) is not allowed.’ [16]^(p.647)^ This then brings into question whether and to what extent current practice is compatible with the CRPD. It has also led to a rise in the number of researchers with lived experience of CTOs and other forms of coercive mental health care examining this issue, either as individuals or in collaboration with others, and a proliferation of advocacy within survivor movements [17, 18].

Australia has some of the highest rates of CTO use internationally. In some jurisdictions, the rate of CTOs may exceed 100 per 100,000 population [9]. While there are significant differences in mental health law across the eight jurisdiction in Australia, CTOs are authorised in broadly similar terms with some safeguards in place in each. Substantial variation in CTO‐rates exists between and within Australian states and territories, suggesting that CTO use is influenced by systemic, cultural, or other factors within mental health services [7, 8, 19]. Differences in practice raise concerns about inequity and inconsistency in decision‐making.

Empirical evidence regarding CTO outcomes is mixed. Evidence from randomised controlled trials has found no evidence that CTOs reduce hospitalisation or improve mental state, social functioning, quality of life, or satisfaction with care [20, 21, 22, 23]. A meta‐analysis similarly found no overall benefit in health service use [6], a conclusion reinforced by an umbrella review of systematic reviews and meta‐analyses covering observational and experimental designs across multiple countries [24]. That umbrella review did, however, determine that improved readmission rates and bed days reported in some studies could be due to CTO use being better targeted towards the people most likely to benefit, such as those with psychosis [24]. A recent retrospective matched case‐control study using linked administrative health data from NSW (2017‐2023) found that CTOs did significantly lower the odds of readmission for individuals with non‐affective psychosis [25]. However, these papers do not address how this is the case, nor do they adequately measure the extent to which assertive voluntary treatment or person‐led care would work better than CTOs.

Although some studies report more favourable outcomes [26, 27], these findings have been challenged on the grounds that CTOs may function as administrative mechanisms that prioritise individuals for service access rather than producing intrinsic therapeutic benefit [28]. Such findings reinforce concerns that CTOs may have become a de facto gateway to care in under‐resourced mental health systems.

Qualitative research provides insight into the complexity of CTO experiences, revealing mixed perspectives. A systematic review of qualitative studies examining consumer experiences identified recurring themes of coercion, limited understanding of CTO processes, variable relationships with clinicians, and ambivalence about perceived benefits and harms [29]. A recent review incorporating perspectives of consumers, carers, and practitioners across several countries internationally found that while views differed, consumers overwhelmingly experienced CTOs as coercive and distressing [10]. Importantly, these views were influenced by consumer trust in clinicians, quality of supports provided, and the nature of therapeutic relationships [10, 29, 30, 31, 32, 33].

Research also highlights a degree of ambivalence about CTOs. For instance, studies from New Zealand and Norway have found that, although consumers frequently experienced CTOs as coercive, dehumanising, and disempowering, many acknowledged perceived benefits, particularly when CTOs were seen as preferable to involuntary hospitalisation or as providing continuity of care [34, 35, 36, 37]. UK research reports mixed perspectives [30, 33, 34]. Haynes and Stroud's cross‐sectional surveys and interviews with mental health professionals and consumers across England and Wales [30] found consumer perspectives to be mixed, though more favourable than adverse; noting though that a large number of consumers declined the invitation to participate, and the dominant consumer perspective being that their CTO was focused on adherence to medication regimens. They also perceived that they had no voice and that care plans did not increase their sense of involvement or engagement; however, they also perceived their relationships with care coordinators as helpful, as they provided ‘evidence of being listened to and cared for’^31(p. 473)^ Canvin et al's study with 75 consumers, carers, and psychiatrists in England and Wales [38] concluded that care experiences varied widely, CTOs are only effective for some people and that shortcomings are rife within the mental health system. Canadian research reported that CTO use may improve consumers' physical health by providing better access to care and closer monitoring [39]. Whereas other Canadian research concluded that CTOs added to ‘already problematic feelings of stigma and powerlessness.’ [40]^(p.48)^

Australian qualitative research mirrors these findings while highlighting specific concerns related to harm, risk, and service culture. Consumers describe CTOs as coercive, traumatic, and iatrogenically harmful, particularly in relation to forced medication, lack of choice, and inadequate communication about medication [31, 41, 42, 43, 44, 45, 46, 47, 48, 49, 50]. A dominant risk‐focused culture within mental health systems is apparent, where clinical decisions are shaped by risk aversion and a hierarchy of knowledge that privileges professional judgement over LLE. [47, 48, 49, 50]. Within this context, coercive responses are common, and consumers report having little voice in care planning, while experiencing CTOs as punitive rather than supportive [43, 49, 50]. Trust (or its absence) has consistently emerged as central to consumers' perceptions of CTOs [31].

Despite jurisdictional differences, there is consistency across international and Australian qualitative literature in the experiences reported by consumers, carers, and practitioners. These similarities include concerns about harm, autonomy, and rights, and point to systemic influences including health service availability and organisational culture, as opposed to clinical factors alone.

Research meaningfully led by consumers and grounded in LLE remains limited. Studies co‐designed, co‐produced, or led by researchers with LLE are rare despite repeated calls for greater consumer involvement in CTO research and policy development [10, 41, 51, 52]. This lack of engagement represents a significant gap in the evidence.

The aim of this study was to address this gap by exploring factors contributing to the use of CTOs in Australia from a consumer LLE perspective. Led and informed by LLE researchers and advisors, the study explored how CTOs are experienced, interpreted, and negotiated by consumers within contemporary mental health systems.

## 2 Methods

### 2.1 Ethics

Ethics approval was obtained from the Flinders University Human Research Ethics Committee (No.6854).

### 2.2 Design and Survey Development

This study was part of a larger project examining factors influencing variation in CTO use in Australia [53]. The project included analysis of mental health policy and legislation; quantitative analysis of administrative data on CTO use; surveys with consumers, family‐carers, and mental health service staff; and qualitative research with each stakeholder group drawn from a small number of sites with high and low CTO use.

The consumer survey reported here used a mixed‑methods design, incorporating Likert‑scales, multiple‑choice, and open‑ended questions to generate quantitative and qualitative data. Survey design enabled coverage of questions examining the continuum of experience of CTOs that were of potential relevance and reflected gaps in the literature to date. It enabled consumer participants to qualify their responses and share their LLE through open‐ended questions. The survey asked participants about their experience with CTOs, including their understanding of when and why a CTO was made. Questions were developed iteratively through a cyclical process which included drawing on existing research evidence [29] on factors that drive the use of CTOs, incorporating input and co‐design with the project's 13‐member LEAP which provided critical insights to design, methods, analysis and dissemination of project findings.

### 2.3 Sample and Recruitment of Participants

A non‐probability convenience sampling strategy plus opportunity snowball sampling method was used to recruit participants. The survey was open to individuals aged 18 and over with LLE of being on a CTO or who self‐identified as facing the threat of being placed on a CTO during their contact with mental health services (henceforth ‘consumers'). Consumer perspectives were collected via an anonymous online survey hosted at La Trobe University using REDCap [54, 55]. The invitation to participate in the survey included a link to the HREC‐approved Participant Information Sheet and electronic consent form. The study information and survey link were promoted to consumers via electronic newsletters and social media through Australian mental health LLE organisations and peak bodies. Survey information was further promoted through extensive networks of the researchers and the project's LEAP, including the project's Aboriginal and Torres Strait Islander Cultural Consultant's network and connections. The survey was open for 6 weeks (July‐September 2024). A AU$10 virtual gift card was offered to participants.

### 2.4 Data Analysis

Both quantitative and qualitative data analysis were used. Due to automated (‘bot’) data contamination of the survey sample (see Supporting File 1), a final sample of 43 survey responses (from the original 1563 responses) was identified as genuine and included for analysis. For quantitative data analysis, R software was used [56]. Due to the small sample size, descriptive analysis was undertaken with results reported as percentages.

Qualitative data was analysed using Latent Content Analysis for open‐ended questions to ensure the structure of key sections and questions could be used to explore the range of issues and themes [57]. Latent Content Analysis is useful for examining LLE and gaining a deeper, interpretive analysis to produce phenomenological descriptions to explain LLE [57, 58]. To code the qualitative data, all responses were read iteratively by EW, SL and TK who independently noted recurring issues and comments and then met to discuss their interpretations of the data. Text responses within each survey question were then coded within the survey content categories for analysis, followed by discussions about coding structure to determine draft overarching and subordinate codes within each category. Descriptive text to capture the findings in each category were then built, and direct quotations that best exemplified the ideas were selected. The LEAP and wider research team reviewed this draft analysis at an early stage and again once further development was undertaken based on their insights. Disagreement of interpretive differences were resolved through robust and respectful discussion that privileged LLE advice from the LEAP. The analysis was shaped by reflexivity and positionality that was cognisant of present and past direct and indirect experiences of CTOs and other coercive practices by members of the LEAP and LE researchers within the project team. Other team members shared and acknowledged the impact of their experience of CTOs from a clinical or legal perspective. This combined input provided diversity of perspectives, challenged researchers to make deeper meaning from the ideas, and enabled robust discussion and finalisation of themes.

### 2.5 Reflexive Statement

This study was conducted by a multidisciplinary team with expertise in psychiatry, social work, LLE, law, and ethics. These diverse perspectives informed the development of the survey, particularly our attention to power, coercion, and culturally safe practice. We recognise that our professional and experiential backgrounds shaped the framing of questions and interpretation of findings, and we sought to mitigate this by using co‑design processes and ongoing reflexive discussion at each stage of the study.

## 3 Results

This section summarises demographic information about the sample. Then, quantitative and qualitative results (exemplified with verbatim deidentified participant quotes – P1, P2, etc) are displayed together according to their organisation around five key aspects of CTO experiences: (1) prior to being placed on a CTO; (2) attitudes towards CTOs; (3) experiences whilst on a CTO; (4) family involvement, and; (5) views on mental health services.

### 3.1 Demographic Information about the Sample

Data from 43 consumers was included in the study. Most participants identified as female (86%), with the largest group aged 30‐49 years (51.2%). Participants were spread across metropolitan, regional and rural Australia in‐line with national population distribution patterns. Most consumers reported that they were not currently on a CTO (92.8%), though many (40.5%) had been on a CTO within the past 1‐5 years, and 45.2% had been on a CTO more than once (one had not been on a CTO but had experienced threat of a CTO). See Table 1.

**Table 1:** Demographic characteristics.

Demographic questions	Response options	*N*	Percentage response
Jurisdiction	NSW	7	16.3
	VIC	21	48.8
	ACT	0	0.0
	SA	2	4.7
	TAS	4	9.3
	WA	4	9.3
	NT	0	0.0
	QLD	5	11.6
	Total	43	100.0
Age	18–29	2	4.7
	30‐49	22	51.2
	50‐69	19	44.2
	70+	0	0.0
	Total	43	100.0
Gender	Male	6	14.0
	Female	37	86.0
	Other	0	0.0
	Total	43	100.0
First Nations	Yes	2	100
	No	0	0.0
	Total	2	100.0
CALD	Yes	5	11.6
	No	38	88.4
	Total	43	100.0
LGBTQIA+	Yes	7	16.3
	No	36	83.7
	Total	43	100.0
Disability	Yes	15	34.9
	No	28	65.1
	Total	43	100.0
Location	Capital	25	59.5
	Regional city	9	21.4
	Rural	7	16.7
	Remote	1	2.4
	Total	42	100.0
Diagnosed with a mental health condition^a^	Yes	42	97.7
	No	1	2.3
	Prefer not to answer	0	0.0
	Total	43	100.0
Agree with diagnosis	Yes	23	54.8
	No	14	33.3
	Prefer not to answer	5	11.9
	Total	42	100.0
CTO status/history	Current	3	7.2
	1‐5 years ago	17	40.5
	6‐10 years ago	4	9.5
	10+ years ago	8	19.0
	Timeframe not stated but not current	10	23.8
	Total	42	100.0
More than once on a CTO	Yes	19	45.2
	No	24	54.8
	Total	42	100.0

^a^ This question was asked because some consumers do not identify with mental health diagnostic labels.

### 3.2 Prior to Being Placed on a Cto

Participants were asked to rate their responses to questions about their awareness, understanding, support, and information prior to being placed on a CTO (see Table 2).

**Table 2:** CTO‐related awareness, support, and information provision prior to being placed on a CTO.

Questions	Response options^a^	*n*	Percentage response
I understood what I needed to do to ***support my own mental health*** before I was placed on a CTO	Strongly disagree	11	25.6
	Disagree	6	14.0
	Neutral	10	23.3
	Agree	8	18.6
	Strongly agree	7	16.3
	Don't recall	1	2.3
	Total	43	100.0
I ***knew about mental health services*** before I was placed on a CTO	Strongly disagree	5	11.6
	Disagree	9	20.9
	Neutral	5	11.6
	Agree	12	27.9
	Strongly agree	11	25.6
	Don't recall	1	2.3
	Total	43	100.0
I was ***aware of my rights*** or was ***given information about my rights*** before being placed on a CTO	Strongly disagree	18	41.9
	Disagree	10	23.3
	Neutral	2	4.7
	Agree	6	14.0
	Strongly agree	5	11.6
	Don't recall	2	4.7
	Total	43	100.0
I was ***provided with legal advice*** in a timely way before being placed on a CTO	Strongly disagree	27	62.8
	Disagree	9	20.9
	Neutral	3	7.0
	Agree	3	7.0
	Strongly agree	0	0.0
	Don't recall	1	2.3
	Total	43	100.0
The doctor/health professional discussed the option of a CTO with me in a way that ***made sense*** to me	S/disagree	19	50.0
	Disagree	11	28.9
	Neutral	2	5.3
	Agree	5	13.2
	S/agree	1	2.6
	Don't recall	0	0.0
	Total	38	100.0
The doctor/health professional discussed the option of a CTO with me in a way that ***made me feel safe***	S/disagree	21	55.3
	Disagree	9	23.7
	Neutral	5	13.2
	Agree	2	5.3
	S/agree	0	0.0
	Don't recall	1	2.6
	Total	38	100.0
I was ***given enough time to consider*** and tell them what I believed I needed.	S/disagree	23	60.5
	Disagree	8	21.1
	Neutral	2	5.3
	Agree	2	5.3
	S/agree	3	7.9
	Don't recall	0	0.0
	Total	38	100.0
I was ***given enough time*** and ***support to understand*** what was happening and what they were saying to me	S/disagree	20	52.6
	Disagree	10	26.3
	Neutral	2	5.3
	Agree	4	10.5
	S/agree	2	5.3
	Don't recall	0	0.0
	Total	38	100.0
The doctor/health professional ***listened*** to me and took my concerns seriously	S/disagree	24	63.2
	Disagree	3	7.9
	Neutral	6	15.8
	Agree	5	13.2
	S/agree	0	0.0
	Don't recall	0	0.0
	Total	38	100.0
The CTO option was ***explained*** to me prior to being made	S/disagree	6	15.8
	Disagree	24	63.2
	Neutral	3	7.9
	Agree	5	13.2
	S/agree	0	0.0
	Don't recall	0	0.0
	Total	38	100.0
I was ***offered other support options*** prior to being placed on a CTO	S/disagree	3	7.9
	Disagree	29	76.3
	Neutral	4	10.5
	Agree	2	5.3
	S/agree	0	0.0
	Don't recall	0	0.0
	Total	38	100.0
I was ***provided with other support options*** prior to being placed on a CTO	S/disagree	5	13.5
	Disagree	27	73.0
	Neutral	3	8.1
	Agree	2	5.4
	S/agree	0	0.0
	Don't recall	0	100.0
	Total	37	
I was ***provided with an interpreter*** to help me understand what they were saying to me	Yes	5	13.2
	No	13	34.2
	Don't recall	0	0.0
	NA	20	52.6
	Total	38	100
I was ***able to have a person I trust*** present when meeting with the doctor/health professional	S/disagree	10	26.3
	Disagree	21	55.3
	Neutral	3	7.9
	Agree	4	10.5
	S/agree	0	0.0
	Don't recall	0	0.0
	Total	38	100.0

^a^ S/disagree = strongly disagree; S/agree = strongly agree; NA = not applicable

Consumers' understanding of what they needed to support their mental health was mixed, as was their knowledge of mental health services. Two‐thirds of participants (65.2%) reported that they were not aware of or given information about their rights, and 63.7% reported they were not provided with timely legal advice prior to a CTO being made. Approximately two‐thirds or more of participants disagreed or strongly disagreed that the CTO was explained in a way that made sense to them (78.9%); in a way that made them feel safe (79%); with enough time to consider and tell health professionals what they needed (62.6%); with enough time and support to understand what was happening (78.9%); and 71.1% disagreed or strongly disagreed that they felt listened to and had their concerns taken seriously.

Consumers were asked about support options available to them, and 76.3% said they were not offered other supports, 73% reported they were not provided with other supports prior to a CTO being made or during the time a CTO was being proposed, and 55.3% reported they were unable to have a person they trusted present when meeting with mental health professionals. Of the participants (n = 18) who responded ‘yes’, ‘no’, or ‘don't recall’ to whether they were provided with an interpreter, 34.2% said they were not. Given that far fewer (11.6%) indicated they identified as culturally diverse or as First Nations (4.7%), potential problems with the question structure may have impacted responses. See Table 2.

### 3.3 Powerlessness in the Face of Clinical Expertise

The theme of powerlessness of LLE expertise within the system alongside clinical and legal expertise was pervasive in the qualitative responses. Participants stated that their voices were not acknowledged, their rights neglected or dismissed, and proper procedures were not observed. Their comments were predominantly negative, conveying a sense that they felt ‘othered’ (marginalised, excluded and dehumanised).

I had no understanding of the reasons that may be used to put me on a CTO. I was not provided with any information regarding legal options, how long the CTO was for.

(P1, female, aged 50–69 years, CTO in the last 5–10 years)

Extremely poor service from medical professionals abusing clinical legislations… disregard to procedures, intimidating tactics used, no privacy regarded in the community, false statement of reasons and not allowing an independent medical professional for assessment within 24 h.

(P2, female, aged 50–69 years, CTO in the last 1–5 years)

During an admission to the ward, I was brought before the mental health tribunal and asked if I agreed to a CTO, not knowing what it was and what it entailed. I was given no information or legal advice, that it was my only way to be discharged from Hospital, therefore agreed.

(P3, female, aged 30‐49 years, CTO in the last 5‐10 years)

### 3.4 Abuse, Trust and Being Lied To

Lack of trust and a sense of being betrayed by health professionals was particularly apparent with the use of the word ‘lied’:

I was forcibly restrained despite no aggression or threat, given Ketamine. Medical Centre then LIED about what was administered and how long I was restrained for.

(P4, female, aged 30‐49 years, CTO in the last 1‐5 years)

A pervasive theme accompanying powerlessness was the feeling of being abused by mental health providers and the system of forced treatment. Comments revealed the impact that feeling ‘abused’ had on the person's mental health.

These orders are akin to being in an abusive relationship and compound the harms experienced by promoting the abuser as the ‘carer’ or sane one.

(P5, female, aged 50‐69 years, CTO across all timeframes (1‐10+ years))

Feelings of being abused were reinforced by reference to feeling threatened and afraid:

CTO was used as a threat or punishment. It was never an agreed or discussed option. It was always forced upon.

(P6, female, aged 18‐29 years, CTO timeframe not specified)

I was constantly threatened with being placed on a CTO if I didn't adhere to what the psychiatrist wanted me to do. I was threatened about having my children taken from me if I didn't comply with psychiatrists demands…The way I was threatened amounted to blackmail. How is that not illegal?

(P7, female, aged 50‐69 years, CTO > 10 years ago)

### 3.5 Attitudes Towards CTOs

Participants were asked to rate their responses to questions about their attitudes towards CTOs. 59.5% believed that CTOs arere not effective. Responses were mixed regarding whether CTOs were less restrictive than inpatient orders. Most participants (75.7%) believed other options should be tried prior to a CTO being decided upon. Most (81.1%) believed that CTOs make the relationship with service providers more difficult. More than half (59.4%) strongly disagreed that CTOs are a useful safety net for people experiencing distress, though 21.6% agreed that they were useful during such times. These findings highlight the complexity of attitudes towards CTOs and the impact on relationships with services for consumers. See Table 3.

**Table 3:** Consumer attitudes towards CTOs.

Questions	Response options^a^	*n*	Percentage response
***CTOs are*** an ***effective*** way to make people have treatment	S/disagree	17	40.5
	Disagree	8	19.0
	Neutral	10	23.8
	Agree	4	9.5
	S/agree	3	7.1
	Total	42	100.0
***CTOs are less restrictive*** on people than inpatient orders	S/disagree	11	26.2
	Disagree	2	4.8
	Neutral	12	28.6
	Agree	14	33.3
	S/agree	3	7.1
	Total	42	100.0
There are **other things that should be tried** before a CTO is decided upon	S/disagree	5	13.5
	Disagree	2	5.4
	Neither	2	5.4
	Agree	9	24.3
	S/agree	19	51.4
	Total	37	100.0
Being on a CTO **can make relationships with service providers difficult**	S/disagree	2	5.4%
	Disagree	1	2.7%
	Neither	4	10.8%
	Agree	9	24.3%
	S/agree	21	56.8%
	Total	37	100.0
CTOs are **a useful safety net** for people who experience mental distress whilst they are receiving treatment/recovering	S/disagree	14	37.8
	Disagree	8	21.6
	Neither	7	18.9
	Agree	6	16.2
	S/agree	2	5.4
	Total	37	100.0

^a^ S/disagree = strongly disagree; S/agree = strongly agree.

### 3.6 Experiences Whilst on a CTO

Participants were asked about their experiences whilst on a CTO. Across all seven questions, participants uniformly expressed negative experiences; 62.2% reported that they were not provided with information on medications to be used as part of the CTO, nor were they involved in selecting these medications (86.5%). Most (81.1%) indicated they were not engaged in decisions pertaining to their treatment and care plans; many (62.2%) reported not receiving a copy of their plans, and a majority (70.2%) were unaware of their plan's existence; 64.9% stated that the service did not listen to them when reapplying for a CTO, and only 13.5% believed their voices were authentically heard at that time. Most (83.8%) characterised the experience of being on a CTO as traumatic. See Table 4.

**Table 4:** Consumer participants' experiences and perspectives of service provision whilst on a CTO.

Questions	Response options^a^	*n*	Percentage response
I was ***given information about medications*** that would be used as part of the CTO	Yes	8	22.2
	No	23	63.9
	Don't recall	3	8.3
	NA	2	5.6
	Total	36	100.0
I ***had a say*** in which ***medication*** **s** I was forced to take	Yes	2	5.6
	No	32	88.9
	Don't recall	1	2.8
	NA	1	2.8
	Total	36	100.0
I ***received a copy*** of the ***treatment and care plan***	Yes	8	22.2
	No	23	63.9
	Don't recall	4	11.1
	NA	1	2.8
	Total	36	100.0
I was ***aware of the treatment and care plan*** that listed what being on a CTO would involve	S/disagree	15	41.7
	Disagree	11	30.6
	Neutral	5	13.9
	Agree	5	13.9
	S/agree	0	0
	Don't recall	0	0
	Total	36	100.0
I was ***involved in deciding*** what the ***treatment and care plan*** would contain	S/disagree	22	61.1
	Disagree	8	22.2
	Neutral	2	5.6
	Agree	3	8.3
	S/agree	1	2.8
	Don't recall	0	0
	Total	36	100.0
Being on a ***CTO was traumatic*** for me	S/disagree	3	8.3
	Disagree	0	0
	Neutral	1	2.8
	Agree	4	11.1
	S/agree	27	75.0
	Don't recall	1	2.8
	Total	36	100.0
The mental health service ***listened to me*** when deciding on whether to reapply for a CTO	S/disagree	19	52.8
	Disagree	5	13.9
	Neutral	4	11.1
	Agree	4	11.1
	S/agree	1	2.8
	Don't recall	3	8.3
	Total	36	100.0

^a^ S/disagree = strongly disagree; S/agree = strongly agree; NA = not applicable

### 3.7 Powerlessness and Experiences of Threat and Abuse of Power By Mental Health Professionals

Participant perspectives on powerlessness prior to a CTO were also reflected in their experiences while on a CTO. The dominance of clinical and legal models of expertise negated individual LLE. Underlying several comments was a lack of faith or trust in the mental health system.

Diagnosis varies each admission to hospital. No definitive diagnosis. Different medications talked about. No confirmatory diagnosis or plan of treatment ever made.

(P8, male, aged 30‐49 years, CTO timeframe not specified)

The pervasive themes of powerlessness and sense of threat or actual abuse were evident in most comments in this section. The impacts of being placed on a CTO were recounted as traumatic and long‐term. How participants felt they were treated while on a CTO severely influenced future relationships with mental health providers.

Traumatic to say the least. It still affects me.

(P4, female, aged 30‐49 years, CTO in the last 1–5 years.)

People think CTOs are less restrictive than ITOs [Inpatient Treatment Orders]. But ITOs are usually shorter‐lived. Back‐to‐back CTOs can go on for years … they can be experienced as more restrictive because you are never free of being forced to take treatment.

(Respondent 9, male, aged 30–49 years, CTO timeframe not specified)

Some participants described how CTOs reinforced existing power structures. As an example, one consumer wrote that:

CTOs are abusive and cause lifelong fear and inability to raise fault with others … because as soon as you raise issues with carers or clinicians, you're seen as troublesome and [are] gaslighted and lied to about your situation. This causes scars that don't seem to heal no matter how old I am getting.

(Respondent 10, female, aged 30–49 years, CTO timeframe not specified)

### 3.8 Positive Experiences of CTOs

A smaller number of participants (*n* = 2) made positive comments; both on a CTO > 10 years ago:

Being on the CTO allowed me space to work on my mental health recovery. I was able to have insight into my mental health issues and being on the depot supported this.

(Respondent 11, female, aged 30–49 years, CTO > 10 years ago)

Without this I possibly would not be here my last attempt was a month after my father's death…they were very understandable and very helpful which is good because it means that they are able to help anyone with problems and time.

(Respondent 12, female, aged 50–69 years, CTO > 10 years ago)

Additionally, some participants commented that when mental health staff worked in partnership with them, their explanations and efforts to build understanding could lessen the coercive nature of the CTO:

When explained to me what it's [CTO] all about and doctors worked with me then they were okay but often it felt like a punishment and that I could not be trusted … if I could see a mental health advocate for the Mental Health Advocacy Service, I felt that I would understand more and what my rights were.

(Respondent 13, male, aged 50–69 years, CTO timeframe not specified)

### 3.9 Family Involvement

Participants were asked questions about their family/supporters' involvement in decisions about the making of a CTO application. Many (60.5%) said health professionals did not include their family/supporters in discussions, and only 23.7% reported their family/supporters were listened to when health professionals were considering applying or re‐applying for a CTO. A notable minority of consumers could not recall whether family/supporters were involved at these times. Only 29% of consumers said their preference for family/supporter involvement was respected by health professionals. See Table 5.

**Table 5:** Family involvement.

Questions	Response options	*n*	Percentage response
The doctor/health professional ***discussed the option of a CTO*** with my family or supporters before the CTO was made	Yes	7	18.4
	No	23	60.5
	Don't recall	8	21.1
	NA	0	0.0
	Total	38	100.0
The mental health service ***listened to my family or supporters*** when deciding on whether to ***apply*** for a CTO	Yes	9	23.7
	No	20	52.6
	Don't recall	6	15.8
	NA	3	7.9
	Total	38	100.0
The mental health service ***listened to my family or supporters*** when deciding on whether to ***reapply*** for a CTO	Yes	9	23.7
	No	19	50.0
	Don't recall	4	10.5
	NA	6	15.8
	Total	38	100.0
***My preference for family involvement*** or ***not*** was respected	S/disagree	6	15.8
	Disagree	6	15.8
	Neutral	11	28.9
	Agree	3	7.9
	S/agree	8	21.1
	NA	4	10.5
	Total	38	100.0

*Notes:* S/disagree = strongly disagree; S/agree = strongly agree; NA = not applicable.

### 3.10 Family Powerlessness and Co‐Option By the Clinical System

Themes of powerlessness and not being heard continued to underlie the comments in this section, related to lack of family/supporter inclusion, lack of respect for the consumer's wishes, and lack of support. Two participants commented that their family was ‘co‐opted’ by mental health staff to be involved in medication compliance. Use of the words ‘monitor’ and ‘police’ reveals how the participant viewed the role assigned to their family:

My family were co‐opted by the service to monitor and police my adherence to neuroleptics.

(Respondent 14, female, aged 50‐69 years, CTO timeframe not specified)

Some participants commented that inappropriate family/supports were involved, contrary to their wishes:

I was gaslighted by my husband and then blackmailed by him and the psychiatrist in cahoots with each other. If I left my husband, I would have lost my children too according to both the psychiatrist and my husband.

(Respondent 7, female, aged 50‐69 years, on more than one CTO > 10 years ago)

Lack of respect for the participant's preferences highlighted how they lacked an active role in decision‐making about their care:

I know the hospital was talking to my mother, but I don't know if they talked about the depots with her. But I need to say that if they did talk to her, this was a not a good thing. My mother helped cover up my child sexual abuse, she was also a perpetrator of physical abuse to me. I did not want her involved because she was part of what had sent me mad in the first place, but I had no say. It's revolting to me that families are involved by services without our express consent when so many of us have been abused by family members. It makes psychiatry complicit in our ongoing abuse.

(Respondent 15, female, aged 50‐69 years, CTO > 10 years ago)

### 3.11 Views on Mental Health Services

Participant responses to questions about the mental health services where they had experience of CTOs were mixed. Most consumers (70.2%) believed that psychiatrists ultimately decided whether a CTO was needed regardless of the views of other mental health professionals in the team. Responses were mixed about whether the balance was right between mental health professionals' direct contact with them versus administrative non‐contact tasks. Their views on the balance being right between community and inpatient/crisis services in their service region were also mixed, though 62.1% believed the balance was not right. Responses were mixed regarding whether mental health professionals act compassionately and whether they trust/believe what consumers say, have time to provide the support people need, and have the skills or resources required to do the job. The final question in this section asked participants' their views on whether people are not followed up unless they are on a CTO. See Table 6.

**Table 6:** Consumer perspectives of the mental health service.

Questions	Response options^a^	*n*	Percentage response
In the MH service that made the CTO, the ***psychiatrists' decision*** about the need for the CTO was final. The views of other mental health professionals weren't really included	S/disagree	3	8.1
	Disagree	2	5.4
	Neither	5	13.5
	Agree	8	21.6
	S/agree	18	48.6
	Don't know	1	2.7
	Total	37	100.0
The ***balance between direct care and contact*** with the person on a CTO and time spent on administrative paperwork tasks is about right in the service I am/was linked to	S/disagree	10	27.0
	Disagree	5	13.5
	Neither	15	40.5
	Agree	2	5.4
	S/agree	1	2.7
	Don't know	4	10.8
	Total	37	100.0
The balance between ***community services and inpatient/crisis services*** is about right in the service region where I live	S/Disagree	17	45.9
	Disagree	6	16.2
	Neither	8	21.6
	Agree	4	10.8
	S/Agree	2	5.4
	Don't know	0	0.0
	Total	37	100.0
In the MH service in my area, health professionals act ***compassion*** **ately** towards people experiencing mental distress	Never/almost never	9	25.0
	Rarely	8	22.2
	Sometimes	9	25.0
	Usually	8	22.2
	Always	2	5.6
	Don't know	0	0.0
	Total	36	100.0
In the MH service in my area, health professionals ***trust/believe*** the information provided by people experiencing mental distress is true	Never/almost never	10	27.8
	Rarely	8	22.2
	Sometimes	12	33.3
	Usually	3	8.3
	Always	2	5.6
	Don't know	1	2.8
	Total	36	100.0
In the MH service in my area, health professionals have the ***time*** to provide the support that people want or need	Never/almost never	12	33.3
	Rarely	8	22.2
	Sometimes	8	22.2
	Usually	5	13.9
	Always	1	2.8
	Don't know	2	5.6
	Total	36	100.0
In the MH service in my area, health professionals have the ***skills*** they need for the job they do	Never/almost never	11	30.6
	Rarely	7	19.4
	Sometimes	8	22.2
	Usually	6	16.7
	Always	2	5.6
	Don't know	2	5.6
	Total	36	100.0
In the MH service in my area, health professionals have enough resources to provide the ***support*** want or need	Never/almost never	8	22.2
	Rarely	11	30.6
	Sometimes	8	22.2
	Usually	3	8.3
	Always	2	5.6
	Don't know	4	11.1
	Total	36	100.0
In the MH service I have most contact with, people are not ***followed up*** for long unless they are on a CTO	Never/almost never	3	8.3
	Rarely	1	2.8
	Sometimes	10	27.8
	Usually	6	16.7
	Always	6	16.7
	Don't know	10	27.8
	Total	36	100.0

^a^ S/disagree = strongly disagree; S/agree = strongly agree; NA = not applicable.

### 3.12 Inconsistent, Inadequate and Uncertain Service Responses

A theme of variability in access to support services was evident in consumer comments about mental health services.

It's true that many people were discharged from hospital with no ongoing support (no CTO). The balance didn't seem right at all ‐ some people like me had far too many ongoing obligations and surveillance under a CTO whereas other people received no ongoing support after a period of acute distress.

(Respondent 16, female, aged 30‐49 years, CTO in the last 1‐5 years)

Others, noting variability in access to care and support, highlighted the importance of a positive organisational culture and adequate resourcing, expertise, and training within mental health services; all of which they perceived as lacking.

The only outcome the mental health services in my area seem to aim for is to inject people with medication and place them on CTOs in order to control them. Apart from this, no other support is offered or given, and nobody speaks to us or our carers about other options, and they never give us a say or listen to our input.

(Respondent 17, male, aged 50‐69 years, currently on a CTO)

In my experience the quality of mental health workers in my area, either have limited MH knowledge or are just not interested. I feel there is a high level of stigma with most of the MH workers in my area.

(Respondent 1, female, aged 50‐69 years, CTO 5‐10 years ago)

## 4 Discussion

In our survey, consumers' experiences of CTOs were found to be predominantly negative, with those experiences often undermining trust in the mental health system and ultimately disempowering consumers in their own mental health care. Participants described being unheard, ignored, disbelieved, and unsupported. Many described being harmed in the short and long‐term by experiences of coercion.

Survey responses indicated that consumers valued and expected to be included in decisions about whether compulsory treatment was necessary. They expressed a desire to be actively involved in discussions about their care both prior to and during a CTO, but they reported being ignored, dehumanised, and unacknowledged when this did not occur. Dawson et al. [50] observed that consumers' knowledge of their own experiences is often dismissed by clinicians or regarded as untrustworthy. Our findings support this observation. As shown in Table 6, only 5.6% of consumers reported that mental health professionals consistently trusted and believed the information provided to them by the consumer.

The survey responses consistently noted that broader sources of support (such as family and supporters) were frequently unacknowledged, not involved in treatment plans, and not given information, even when consumers explicitly requested that their networks be involved in their care. Conversely, for some, family members were involved despite the consumer's express wishes for this not to occur. Survey data presented in Table 5 indicate that consumers experienced their support networks being either left out of conversations or, as one consumer noted, ‘co‐opted’ by the clinical care teams to monitor their behaviour. These findings support Corring et al's [59] review of 12 qualitative studies examining the experiences of the families of people on a CTO, which reported that, while many families found CTOs to be beneficial, others' experiences mirrored the consumer responses in our survey, which emphasised that CTOs are perceived to be overly concerned with compliance and adherence over the person's more holistic needs. More recently, Karanikolas^1(p.3)^ described CTOs as part of a ‘psychiatric circuit’ involving surveillance, coerced mobility, routine police involvement, and ‘transcarceral’ transfers into police and family‑policing systems. It explicitly frames the everyday operation of CTOs as drawing families into coercive governance structures rather than therapeutic support.

The role of service culture, service structure and resources available to mental health services appeared to impact perceptions about the type of care that clinicians offer to consumers. While some consumers acknowledged wider factors affecting their care (e.g., adequate resourcing, expertise, and training for mental healthcare workers), many expressed that these were not excuses for poor care. Study participants reported experiencing significant stigma and control within services, focused on compliance. Dawson et al. [50] found that ‘individual workers were constrained by the dominant service culture, which endorsed care that was task‐based’ (p. 311),for example, medication compliance or assessment of risk, rather than sustained therapeutic arrangements. Santamaría‐García et al [59] assessed different dimensions of empathy by mental health clinicians compared with general‐physicians and non‐clinicians. They found that mental health clinicians exhibited higher empathic concern and discomfort for others' suffering; however, they were the only group where empathy variability was explained by moral judgements, years of experience (more experienced workers had lower empathy), and workplace type (higher empathy in community *vs.* hospital settings). They concluded that empathy by mental health clinicians may be particularly affected by contextual factors such as poor‐quality care environments and service culture, time pressures, and a progressive desensitisation to psychological and mental suffering.

Examining CTOs from a human rights perspective, Newton‐Howes and Ryan [9] note that workforce and service resource deficiencies within mental health systems are significant determinants of CTO decision‐making, who gets access to mental health support, and how consumers are involved in decision‐making about their care. Participants in our study highlighted significant variability in access to support. Gibb et al's [33, 34] research and its underscoring of ambivalence aids in understanding how people can experience treatment as coercive, yet coalesce, given the perceived lack of alternatives to CTOs in terms of support in the community. This profound ambivalence [33] about CTO use is also emphasised by Light et al's [45, 46, 60, 61] research, which showed that consumer, family, and clinician participants believed CTOs were used to ensure access to services (but still could not remedy inadequate or non‐existent services). Such a system can have adverse implications for how it works with people while they are on a CTO to enhance recovery and help the person to build self‐agency; particularly what is done to support them to not be placed on a further CTO in the future [42, 46]. Support for CTOs, particularly based on clinical and legal decision‐maker views, can be explained by believing that a CTO will provide a safety net and maintain a relationship between services and consumers. However, the experience of our study participants highlights complexity in this narrative and suggests that the impact of CTOs on consumers can actually undermine service engagement.

Building on Dawson et al's [50] ethnographic research on the dynamics of consumer engagement and involvement in decision‐making about CTOs within care planning, our results also provide further insights into how the hierarchy of knowledge access [62] plays out. Our study participants clearly stated that they were not provided with access to information about their rights or alternative support options prior to being placed on a CTO. Furthermore, they emphasised how this dismissal of their part in their own care led to long‐term trust breakdown with mental health care services, clinicians, and their wider networks of support. Similar concerns were noted by Maylea et al. [48] who examined experiences of the Victorian Mental Health Act and found that many consumers were not informed of their rights and not involved in decisions about treatment. Of note, systematic review and meta‐analysis research by De Jong et al. [63] on interventions to reduce compulsory hospital admissions found that use of advance statements (consumer‐stated treatment and care preferences for if and when they become unwell) was statistically significantly associated with reduced compulsory admissions. They proposed that advance statements may therefore be more effective than CTOs. Other research [61] has found that, while CTOs can increase consumers' access to mental health services, they do not offer redress for non‐existent or under‐resourced services. Our findings support this view that CTOs may be being used inappropriately to ensure follow‐up.

Trust, as highlighted by McMillan et al. [31], is a key prerequisite for the empowerment required in a recovery‐oriented approach to mental health care. Across both our qualitative and quantitative findings, trust surfaced as a key theme. Trust emerged as a broad, multifaceted aspect of being placed on a CTO. Some consumers felt that their lack of involvement in their own care reflected that care services and professionals did not trust them. Consumers' responses across the survey emphasised that trust, in various forms, underpins the positive or negative long‐term impact CTOs have on individuals. This finding is supported by the smaller number of consumer respondents who reported positive interactions with practitioners and medical staff because, as they explained, they were ‘included’ and ‘worked with’ in the discussion of their own care. This raises questions about how to also learn more about what creates these more constructive experiences [50].

Some participants reported that they engaged with mental health services or followed treatments primarily due to fear of being placed on a CTO, or because they were explicitly threatened with one. This use of fear, intimidation, implicit coercion, and threats reflects how CTOs (or the threat of a CTO) are viewed as tools of carceral control. Although mental health care has shifted from institutionalisation to community‐based approaches, research [1] indicates that CTOs serve as mechanisms for governance and surveillance. Consumers reported feeling constantly watched, restricted, and told what to do, often fearing further disciplinary measures if they did not comply. For some consumers, the impact of this carceral control in the ‘psychiatric circuit’ [1] was long‐term and created far‐reaching distrust in services, especially regarding future interaction with mental health services. Further, rather than providing a less restrictive alternative to inpatient involuntary treatment, CTOs led to the person's heightened visibility to other carceral state systems (e.g., police, child welfare) beyond mental health services [1]. Research on coercion and pressure in the broader mental health treatment area [1, 64] finds that coercion ranges from the use of persuasion and treatment pressure, to threats, and suggests more work needs to be done in fully defining what coercion and other related terms like ‘exploitation’ and ‘deception’ are in mental health care, how they can be avoided, and what supports and skills mental health clinicians need in order to achieve alternatives to coercion [65, 66].

Of note, a majority (81%) of participants in our study answered that CTOs were traumatic, and this aligns strongly with the existing literature [10, 44] In addition to harms associated with the undermining of trust and help‐seeking for mental health, which have been noted in the existing research [31], further traumatising consumers who may already have experienced trauma raises significant ethical and moral concerns about the costs and benefits of CTO use. Wergeland et al. [67] explored consumer experiences after they had their CTO cancelled and noted that being assessed as having capacity had enhanced consumers' autonomy, their interactions, and feelings of being respected in encounters with health professionals. However, previous experiences of coercion left several participants feeling fearful and passive, which adversely impacted their mental health recovery in the longer term.

This study explored the experiences and perspectives of individuals with LLE of CTOs. Given that experiences of coercion within risk‐focused service cultures are consistently reported by many consumers during contacts with mental health services, regardless of consumers' legal status [65], our findings reflect concern about processes of engagement that are likely relevant and important to all people in contact with mental health services, and to the mental health workforce. As Harvery et al's [10] review concluded, ‘consumer experiences of CTOs depend critically upon the relationship between what is done and how it is done’.^(p.70061)^ Controversially, when Australia ratified the CRPD in 2008, it posted a declaration that it would interpret the CRPD as permitting fully substituted decision‐making as a last resort [12, 13]. A subsequent report by the Australian Law Reform Commission [68] emphasised the importance of supported decision‐making. Most jurisdictions have since revised their mental health legislation with greater emphasis on support and dignity of risk. Introduction of supported decision‐making and a greater focus on dignity of risk and human rights (including development of a human rights tool to support implementation) [66] exemplify this shift. The current research highlights that, despite these more recent positive developments and attempts to improve the situation, consumers' negative experiences of CTOs persist.

### 4.1 Strengths and Limitations

We identified several strengths and limitations of this research. A key strength was the LLE involvement in the research. Another key strength was the number and types of questions posed to participants across the CTO continuum, which yielded rich, extensive data. However, we acknowledge that the length of the survey may have limited accessibility for some potential participants.

The small sample size precluded analysis of more distinct perspectives. Another potential limitation was that several participants had been on a CTO > 5 years ago. We prioritised respect for participants' LLE regardless of whether they had experience or perceived being threatened with a CTO and regardless of the passage of time.

Recruitment was adversely impacted by bot infiltration, which posed a methodological limitation. Of 1,563 survey submissions (excluding those left entirely blank or otherwise unclassified *n* = 70), 9.6% were likely fraudulent, while a further 83.2% were clearly fraudulent or unverifiable (see Supporting File 1). Most participants were recruited through distribution of recruitment information via the broader networks of those involved in the wider project. While not definitive, this recruitment method could have resulted in participants who were more politically active in systemic advocacy, potentially introducing bias in the responses.

Most survey respondents (86%) identified as women. This disparity could reflect the sector in which the information was distributed (i.e. Australian mental health LLE advocacy may engage a higher proportion of women). A meta‐analysis of online survey response rates indicates that women's participation rates were much higher than for men [69]. However, sociological research into the recorded yet underexplored disparity in survey responses by gender identity has found that, although more women respond, the reason for this disparity remains inconclusive [70].

The largest group of participants were from the Australian state of Victoria (48.8%). Concentration of responses from one jurisdiction may have shaped themes and trends identified, speaking more specifically to the Victorian context. Furthermore, it could make the research's generalisability more difficult. However, a strength of the study is that participating consumers were located throughout remote, rural, regional, and metropolitan areas. This geographic diversity captures variation in services and service access broadly, allowing consideration of different care contexts, infrastructure, and CTO implementation. Previous research has found that CTO use in Australia varies both across and within states in Australia [8].

A final limitation with respect to demographic particulars, is the underrepresentation of participants from culturally and linguistically diverse (CALD) backgrounds (11.6%), relative to the national population born overseas (28%) according to the national census [71]. Other contemporary research concludes that individuals born outside of Australia and those of CALD backgrounds are more likely to be placed on CTOs [1, 72], Hence, we suggest future research needs to develop more targeted strategies to recruit people from CALD backgrounds.

## 5 Conclusions

This research shows that, while policy and research have continued to examine CTO use, little has changed over time for consumers in relation to CTOs. Our findings reflect that mental health care associated with CTO use is still largely experienced by consumers as stigmatised, and CTOs as coercive and often traumatising. The continued lack of continuity of care across mental health care facilities and jurisdictions negatively impacts consumers' experiences, while many also report that exclusion of family and support networks during their care results in worse outcomes. While Australian mental health services aspire to implement supported decision‐making [39], we found that consumers feel left out of their own care and ignored in discussions about CTO implementation and medication regimes. Many of these findings are consistent with earlier research presented here, suggesting that the ways in which service providers engage with people regarding CTOs remain largely unchanged. This persists despite significant efforts and advocacy to reform Australia's mental health system, elevate human rights, improve responses to minimise restrictive practices, and support the growth of the LLE peer workforce. It appears that these efforts have yet to translate into meaningful improvements in consumer engagement and experience. More research is needed particularly in relation to the experience of CALD communities [72], and the factors that improve people's experience in ways that reduce the use of CTOs and/or provide protections from the harms that can result from being on a CTO.

Several specific recommendations are apparent and are reflected in existing international literature, evidence and advocacy [14, 73, 74, 75, 76]. For example, Patel et al. [74] outline 5 principles for transforming mental health systems: 1. Target harmful social environment across the life course to prevent mental health problems and promote mental health; 2. Determine care by a person's needs, not their diagnosis; 3. Empower frontline workers to deliver evidence‐based psychosocial interventions; 4. Embrace a rights‐based perspective in mental health care; 5. Place people with lived experience at the centre of the care system, including stronger investment and integration of lived experience peer workforce across all systems. These principles are currently informing policy and practice reform efforts in Australia and elsewhere; they just need to be implemented meaningfully in practice, with attention to prevention, diversity and equity in care system redesign, more and wiser investment (particularly in community supports for unmet need), and greater accountability [74]. These include robust models of care as alternatives to CTOs, better resourced voluntary community‐based support models, improved and more accountable clinician training and supervision (especially in relation to understanding and delivery of trauma‐informed care and supported decision‐making) [77], and potential legislative reforms that improve how services and their staff operationalise human rights concepts related to capacity assessments, dignity of risk, consumer advance statements, and supported decision‐making.

## Author Contributions

**Sharon Lawn:** conceptualisation, investigation, funding acquisition, writing – original draft, methodology, writing – review and editing, formal analysis, project administration, data curation, supervision. **Tessa‐May Zirnsak:** investigation, methodology, writing – review and editing, formal analysis, data curation. **T. J. Spencer:** investigation, writing – review and editing, methodology, formal analysis, data curation. **Elaine Waddell:** investigation, formal analysis, writing – review and editing. **Jane Fischer:** formal analysis, writing – review and editing, data curation. **Tarmia Klass:** formal analysis, writing – review and editing. **Puneet Sansanwal:** investigation, writing – review and editing, formal analysis. **Edwina T. Light:** funding acquisition, writing – review and editing, methodology, conceptualisation, investigation. **Vrinda Edan:** investigation, funding acquisition, writing – review and editing, methodology, conceptualisation. **Chris Maylea:** conceptualisation, funding acquisition, methodology, writing – review and editing. **Penelope Weller:** conceptualisation, funding acquisition, writing – review and editing, methodology. **Morgan Gould:** writing – review and editing, methodology, resources. **Christopher J. Ryan:** conceptualisation, funding acquisition, methodology, writing – review and editing. **Giles Newton‐Howes:** conceptualisation, funding acquisition, methodology, writing – review and editing. **Steve Kisely:** conceptualisation, investigation, funding acquisition, writing – review and editing, methodology. **Lisa Brophy:** conceptualisation, investigation, funding acquisition, writing – review and editing, methodology, project administration, supervision.

## Ethics Statement

Ethics approval was obtained from the Flinders University Human Research Ethics Committee (No. 6854). All participants provided electronic consent prior to commencing the online survey. All participants provided electronic consent for publication prior to commencing the online survey.

## Conflicts of Interest

The authors declare no conflicts of interest.

## Supporting information


Supporting File

## Data Availability

The data that support the findings of this study are available on request from the corresponding author. The data are not publicly available due to privacy or ethical restrictions.
