# Supplementary material for: ‘Traumatic to Say the Least; It Still Affects Me’: Consumer Perspectives on Community Treatment Orders in Australia
Source: Health Expect. 2026 Jul 17;29(4):e70770. doi: 10.1111/hex.70770 (PMC13378099; doi:10.1111/hex.70770)
Supplement: Supplementary file 1 — Supporting File [file HEX-29-e70770-s001.docx]

**Supplementary File 1: Management of BOT Survey Issue**

Each completed survey was assessed using the screening tool and then ranked based on the likelihood of whether the data had been provided by a real participant. This process was led by two team members (SH and TS) with other authors supporting assessment where there was a disagreement between rankings (LB and TZ). The definitions of our ranking bands can be found in Table 1.

**Table 1: Ranking definitions for cleaned data**

| **Level** | **Definition** | **Markers** |
| --- | --- | --- |
| A | Genuine | Contains qualitative responses to open-ended text box responses that are relevant and make sense, have minimal or no inconsistencies across individual questions. |
| B | Likely genuine | Has no markers characteristic of fraudulent activity but has no or limited qualitative responses to open-ended questions. |
| C | Indeterminate | Has no markers of being fraudulent but no individual answers that indicate a real person had completed this survey. |
| D | Likely fraudulent | Has some markers to indicate fraudulent activity but could still be a genuine response with minor errors (e.g. invalid postcode, but no other markers of AI). |
| E | Fraudulent | Contains two or more of the indicators of AI generated responses outlined above in Table 1. |

Of the 1563 responses to consumer survey (excluding 70 surveys left completely blank or otherwise uncategorised), 39% were found likely fraudulent and 7.8% indeterminate/unverifiable. Only 4.5% were likely genuine responses, and 2.7% were found to be genuine. Table 2 shows the breakdown of responses for the consumer survey.

**Table 2: Consumer survey ranking (1,563 total responses)**

|  | A-Level responses | B-Level responses | C-Level responses | D-Level responses | E-Level responses | Blank or uncategorised |
| --- | --- | --- | --- | --- | --- | --- |
| Count (%) | 43  (2.7%) | 28  (1.8%) | 122  (7.8%) | 609  (39.0%) | 691  (44.2%) | 70  (4.5%) |

Table 3 shows the comparison of the aggregated results to determine the likelihood that the B- and C-Level samples were genuine.

**Table 3: Demographic Data by Ranking**

|  | | **A** | | **B** | | **C** | |
| --- | --- | --- | --- | --- | --- | --- | --- |
|  |  | **n** | **%** | **n** | **%** | **n** | **%** |
| Jurisdiction | NSW | 7 | 20.6 | 6 | 28.6 | 33 | 29.7 |
|  | VIC | 12 | 35.3 | 5 | 23.8 | 21 | 18.9 |
|  | ACT | 1 | 2.9 | 1 | 4.8 | 2 | 1.8 |
|  | SA | 5 | 14.7 | 3 | 14.3 | 10 | 9.0 |
|  | TAS | 2 | 5.9 | 1 | 4.8 | 6 | 5.4 |
|  | WA | 3 | 8.8 | 1 | 4.8 | 12 | 10.8 |
|  | NT | 0 | 0.0 | 0 | 0.0 | 0 | 0.0 |
|  | QLD | 4 | 11.8 | 4 | 19.0 | 27 | 24.3 |
|  | Total | 34 | 100.0 | 21 | 100.0 | 111 | 100.0 |
| Age | 18-29 | 1 | 2.9 | 1 | 4.8 | 4 | 3.6 |
|  | 30-49 | 9 | 26.5 | 14 | 66.7 | 66 | 59.5 |
|  | 50-69 | 18 | 52.9 | 5 | 23.8 | 39 | 35.1 |
|  | 70+ | 6 | 17.6 | 1 | 4.8 | 2 | 1.8 |
|  | Total | 34 | 100.0 | 21 | 100.0 | 111 | 100.0 |
| Sex | Male | 3 | 9.1 | 8 | 40.0 | 48 | 43.2 |
|  | Female | 30 | 90.9 | 11 | 55.0 | 62 | 55.9 |
|  | Other | 0 | 0.0 | 1 | 5.0 | 1 | 0.9 |
|  | Total | 33 | 100.0 | 20 | 100.0 | 111 | 100.0 |
| First nations | No | 34 | 100.0 | 20 | 95.2 | 74 | 67.3 |
|  | Yes | 0 | 0.0 | 1 | 4.8 | 36 | 32.7 |
|  | Total | 34 | 100.0 | 21 | 100.0 | 110 | 100.0 |
| CALD | No | 31 | 91.2 | 16 | 76.2 | 97 | 87.4 |
|  | Yes | 3 | 8.8 | 5 | 23.8 | 14 | 12.6 |
|  | Total | 34 | 100.0 | 21 | 100.0 | 111 | 100.0 |
| LGBTQIA+ | No | 29 | 85.3 | 19 | 90.5 | 110 | 99.1 |
|  | Yes | 5 | 14.7 | 2 | 9.5 | 1 | 0.9 |
|  | Total | 34 | 100.0 | 21 | 100.0 | 111 | 100.0 |
| Disability | No | 26 | 76.5 | 19 | 90.5 | 110 | 99.1 |
|  | Yes | 8 | 23.5 | 2 | 9.5 | 1 | 0.9 |
|  | Total | 34 | 100.0 | 21 | 100.0 | 111 | 100.0 |
| Location - supporter | Capital | 23 | 71.9 | 8 | 40 | 60 | 55 |
|  | Elsewhere | 9 | 28.1 | 12 | 60 | 49 | 45 |
|  | Total | 32 | 100 | 20 | 100 | 109 | 100 |
| Location - consumer | Capital | 21 | 63.6 | 8 | 42.1 | 39 | 35.1 |
|  | Elsewhere | 12 | 36.3 | 11 | 57.9 | 72 | 64.8 |
|  | Total | 33 | 99.9 | 19 | 100 | 111 | 99.9 |
